# What Can Genetics Do for the Control of Infectious Diseases in Aquaculture?

**DOI:** 10.3390/ani12172176

**Published:** 2022-08-25

**Authors:** Simona Sciuto, Licia Colli, Andrea Fabris, Paolo Pastorino, Nadia Stoppani, Giovanna Esposito, Marino Prearo, Giuseppe Esposito, Paolo Ajmone-Marsan, Pier Luigi Acutis, Silvia Colussi

**Affiliations:** 1 Istituto Zooprofilattico Sperimentale del Piemonte, Liguria e Valle d’Aosta, Via Bologna 148, 10154 Torino, Italy; 2 Department of Animal Science, Food, Technology–DIANA, and Nutrigenomics and Proteomics Research Center–PRONUTRIGEN, Università Cattolica del Sacro Cuore, Emilia Parmense 84, 29122 Piacenza, Italy; 3 Associazione Piscicoltori Italiani (API), Via del Perlar 37/A, 37135 Verona, Italy

**Keywords:** aquaculture, infectious disease, pathogen evolution, selective breeding

## Abstract

**Simple Summary:**

Infectious diseases place an economic burden on aquaculture and a limitation to its growth. This state-of-the-art review describes the application of genetics and genomics as novel tools to control infectious disease in aquaculture.

**Abstract:**

Infectious diseases place an economic burden on aquaculture and a limitation to its growth. An innovative approach to mitigate their impact on production is breeding for disease resistance: selection for domestication, family-based selection, marker-assisted selection, and more recently, genomic selection. Advances in genetics and genomics approaches to the control of infectious diseases are key to increasing aquaculture efficiency, profitability, and sustainability and to reducing its environmental footprint. Interaction and co-evolution between a host and pathogen can, however, turn breeding to boost infectious disease resistance into a potential driver of pathogenic change. Parallel molecular characterization of the pathogen and its virulence and antimicrobial resistance genes is therefore essential to understand pathogen evolution over time in response to host immunity, and to apply appropriate mitigation strategies.

## 1. Introduction

Aquaculture is a vital source of high-value protein for human nutrition and plays a central role in the global food economy [1]. The top countries for aquaculture production in Europe are Spain, France, the United Kingdom, Italy, Greece, and Norway. Aquaculture production consists mainly of mussels, salmon, trout, oysters, sea bream, sea bass, and clams.

The fish farming sector in Italy had a turnover of EUR 300 million in 2021, with 62,150 tons of twenty different fish species farmed in over 600 fisheries. Rainbow trout (*Oncorhynchus mykiss*) ranked first by production volume (37,000 tons), followed by sea bream, sea bass (over 17,000 tons), and brook trout (*Salvelinus fontinalis*). The rainbow trout market and its farming in inland waters (Italy is an EU leader) is increasingly oriented towards sizes of over 500 g, a substantial part (>10/15%) of which is devoted to the production of large trout (>1.2 kg) [2].

There is a growing demand for fish products because of their high nutritional value and unsaturated fatty acids. Wild fish catches are decreasing as stocks decline because of overfishing, climate change, and water pollution, whereas aquaculture production has increased dramatically in the last two decades [3]. Indeed, fish farming will play a growing role in meeting the nutritional needs of the human population.

Infectious diseases reduce the economic return of aquaculture production and are a serious problem in the management of aquaculture production, particularly where high-density rearing conditions promote the spread of infectious diseases and induce stress and higher disease susceptibility [4]. In addition, rising global water temperatures and water levels due to climate change threatens aquaculture production and alters disease dynamics [5].

As documented for other livestock production systems, the success and sustainability of aquaculture depends largely on disease control to reduce economic losses due to high mortality and treatment. Reduced disease occurrence and severity are essential to ensure aquaculture productivity, profitability, and efficiency, as well as the welfare of cultured freshwater and marine species.

This review describes the state of the art approaches to infectious disease control in fish farming, from conventional methods to novel genetics and genomics applications, with the aim of improving appropriate mitigation strategies.

## 2. Conventional Disease Control Methods

Conventional disease control relies largely on vaccination and drug treatment. Commercial vaccines are currently available for most, but not all aquatic diseases [6]. Autogenous vaccines obtained from local pathogen strains are employed for limited application on farms where a pathogen is isolated [7]. Vaccine efficacy is often time-limited, depending on the formulation (attenuated or adjuvated), does not cover the entire breeding period, and requires boosters. Efficacy has been reported to decrease with time due to strain evolution and vaccine-escaping mechanisms [8]. Vaccination incurs considerable costs in disease management, and it is also time-consuming, especially for small-scale farming systems, where the costs are spread over a smaller number of doses, leading to high per-unit production costs.

The worldwide increase in the use of therapeutics in aquaculture has increased noticeably during the past two decades, coinciding with changes in farming systems and the intensification of production. The intensive use of antimicrobials may select drug-resistant bacteria that may spread resistance genes to fish and human pathogens, with a negative impact on aquatic ecosystems and human health [9,10]. To mitigate the impact of infectious diseases on aquaculture production, other strategies that can complement existing ones are needed to increase the efficacy of interventions and minimize the risk of environmental pollution. In this context, genetic improvement is a sustainable option as it can increase resistance to infectious disease and reduce the use of antimicrobials.

## 3. Breeding for Disease Resistance in Fish

Selective breeding is playing an ever-important role in aquaculture production as it induces cumulative and permanent improvement in the traits included in selection objectives. Breeding for resistance offers advantages to aquaculture production by increasing efficiency, profitability, and sustainability, and reducing the environmental footprint [11,12,13]. While it has been successfully applied in aquaculture to reduce disease incidence, only a small share of aquaculture production is currently derived from selectively bred stocks. The proportion is increasing rapidly, particularly for species of high economic value, such as crustaceans and mollusks [14].

Methodological and technological advances have progressed from mass selection to quantitative genetics and breeding methods, to candidate gene and quantitative trait loci (QTL) information, and genomic selection. With the very recent introduction of genomics, there is the enhanced potential to enhance and speed up genetic improvement programs and improve production systems based on a more accurate estimate of the relationship between animals, to discover markers in close linkage disequilibrium with genes of biological and economic importance, and to increase the reliability of breeding value estimates, particularly for low heritability traits [14]. Nevertheless, given the host–pathogen interaction and co-evolution, breeding for disease resistance is a potential driver of pathogen evolution to overcome resistance mechanisms [15]. Therefore, changes in the pathogen need to be monitored for its molecular profile, virulence, and antimicrobial resistance.

### 3.1. Conventional Selection

Individual selection for growth has long been applied to aquatic species; however, the use of a small number of breeders without parentage records did not allow for the scientific management of inbreeding.

The high prolificity of aquatic species has made it possible to apply family-based selection to control diseases in aquaculture [16]. Experimental challenge tests on families have shown that disease resistance is a heritable trait with very variable heritability values: from 0.04 to 0.62 for bacterial diseases and from 0.04 to 0.79 for viral diseases [17,18]. This type of stock selection has been widely used in Northern Europe to obtain generations of infectious pancreatic necrosis (IPN)-resistant salmon [19]. IPN is a viral disease of primary concern for salmon farming, with frequent outbreaks responsible for high mortality in stocks. After resistance to IPN was shown to be heritable (h^2^ = 0.38), breeding companies began applying family-based selection programs [14,18], which requires careful management of family stocks to avoid inbreeding and loss of genetic variability.

### 3.2. Candidate Genes and Marker Assisted Selection

In the late 1990s, genetic-based methods began to appear alongside the phenotypic approach and a variety of genetic markers for aquaculture species were developed (Box 1). Marker-assisted selection (MAS) allows for the improvement of traits of interest, such as disease resistance, by selecting for genetic variants associated with resistance through the use of nearby genetic markers. As resistance traits are often complex in nature and causative variants can remain undetected, selection targets a genomic region, termed a quantitative trait locus (QTL), that may comprise several genes. MAS has been applied to viral, bacterial, and parasitic diseases in salmonids [20].

Family-based methods for IPN control were integrated by selective breeding of major QTL mapping on chromosome 21 [21,22,23]. MAS reduced IPN outbreaks by 75% and is now being applied in Scottish and Norwegian breeding programs to achieve genetic resistance to IPN. A major QTL was also identified for resistance to viral hemorrhagic septicemia virus (VHSV) infection, one of the most extensively studied viral diseases in fish [24].

Programs for inducing resistance to bacterial and parasitic diseases based on QTL have been applied to rainbow trout as well. A QTL region was found on chromosome 16 that carries single nucleotide polymorphisms (SNPs) associated with resistance to furunculosis caused by *Aeromonas salmonicida,* which is responsible for severe disease in salmonid aquaculture systems [25]. Major QTLs were found associated with resistance to *Vibrio anguillarum* and *Flavobacterium psychrophilum* [26,27]. Evenhiuset et al. [28] and Silva et al. [29] reported on the efficacy of selective breeding for *Flavobacteria* and resistance to bacterial cold-water disease (BCWD) and columnaris disease (CD), caused by *Flavobacterium psychrophilum* and *F. columnare*, respectively.

Robledo et al. [30] reported on two QTLs on chromosome 18 and an SNP (located at the distal end of chromosome 16, associated with resistance to *Neoparamoeba perurans,* an amoeba very frequent in European salmonid farms and a cause of amoebic gill disease (AGD). Robledo et al. [31] characterized a QTL for host resistance in a population of Caligus-challenged Atlantic salmon. A study on a QTL linked to susceptibility to copepods (*Lepeophtheirus salmonis*) [32] carried out negative selection on the eggs to decrease the QTL frequency in commercial populations.

Salmonids are among the foremost aquaculture species for which selective breeding for disease resistance has been improved. Interesting results in shellfish have also been obtained in control of the white spot syndrome virus (WSSV) that heavily affects shrimp production. Robinson et al. [33] identified several linkage groups containing QTL associated with death after WSSV infection in black tiger shrimp.

However, because QTLs explain only a small part of phenotypic and genetic variance in many cases, conventional MAS tends to be inefficient. When selection is based on candidate or causative genes, it is termed gene-assisted selection (GAS). Studying IPN resistance in salmon, Moen et al. [34] identified the epithelial cadherin gene as the causative locus of resistance to this disease, while Pavelin et al. [35] described the association between inhibition of the NEDD-8 activating enzyme 1 (NAE1) and a high reduction in IPN replication.

The candidate gene approach numbers among the strategies for genetic dissection of disease resistance traits. The candidate gene theory states that a significant proportion of the phenotypic variant of a trait can be ascribed to polymorphisms within genes known to be involved in the physiological regulation of the trait. In salmonids, for example, genes of the major histocompatibility complex (MHC) have been well characterized and reported to be associated with resistance to various diseases. In rainbow trout, Colussi et al. [36] described the MHC class II B-1 domain gene as a candidate for resistance to lactococcosis, an infectious disease caused by the bacterial pathogen *Lactococcus garvieae*. Johnson et al. [37] found a suggestive association between MHC I and resistance to BCWD in rainbow trout. Combinations of MHC I and II were found to significantly influence disease resistance to infectious salmon anemia [38], furunculosis [39], and infectious hematopoietic necrosis virus in Atlantic salmon [40].

Candidate gene-based studies have also defined a variation in a serine protease inhibitor associated with resistance to *Perkinsus marinus*, a pathogenic dinoflagellate in the eastern oyster (*Crassostrea virginica*) [41].

In brief, MAS and GAS are efficient in selecting traits controlled by a few genes or in which few variants explain a substantial portion of the trait’s genetic variance. When the trait is highly multigenic, a different approach can be taken that uses the information provided by myriad markers spread along the genome (genomic selection) [42].

Box 1 **DNA-based methods for the development of genetic markers in aquaculture species.**

**AFLP**
Amplified Fragment Length PolymorphismsRestriction enzymes are used for genomic DNA digestion. A subset of the restriction fragments is selected for amplification by primers complementary to the ligation adaptor sequence, the restriction site sequence, and a few nucleotides inside the restriction site fragments. The markers are a cost-effective alternative in species for which economic resources are limited.
**RAPD**
Random Amplified Polymorphic DNAThe genome is amplified using several arbitrary short primers (10–12 nucleotides). AFLP markers are usually preferred to RAPD because of their greater reproducibility.
**RFLP**
Restriction Fragment Length Polymorphic DNAGenomic DNA is digested by restriction enzymes; the fragments separate on agarose gel and create different patterns. The markers are poorly polymorphic, however, which is a major drawback.
**SSR/STR/VNTR**
Microsatellite RepeatsSpecific sequences of DNA containing tandem repeats. The number of repeats differs for alleles at a specific locus; a specific set of primers is used in simplex or multiplex PCR for loci amplification. These markers are commonly used because of their high polymorphic information content and their wide distribution throughout the genome.
**ESTs**
Expressed Sequence TagsESTs derived from c-DNA libraries, constructed using mRNA expressed in tissues. They are useful tools for marker development in species where the full genome is not yet available.
**SNP**
Single Nucleotide PolymorphismDNA sequence variations at a single nucleotide level are used as genetic markers. They are the most frequent polymorphism in any organism, adaptable to automation, and reveal hidden polymorphisms not detected by other methods.
**ddRAD**
Double-Digest Restriction-Site-Associated DNA SequencingThis method is based on the enzymatic digestion of the whole genomic DNA and the creation of multiplexed libraries, with consequent binding to specific adapters (reduced representation libraries) which are more laborious and less accurate than SNP analysis.

### 3.3. Genomic Selection

Recent studies in genome analysis (e.g., markers, SNP arrays, next generation sequencing technologies (NGS)) have mapped dense markers across the entire genome and estimated the genetic merit of each chromosome fragment contributing to variation in a population with phenotypic observations. Studies have employed SNP markers in genetics and genomics research due to their abundance, relative ease of high-throughput discovery and typing, and low cost of genotyping. A recent study [43] showed that genomic selection in salmonids can be implemented using low-density SNP panels associated with genotype imputation strategies.

Genomic selection relies on genome-wide markers for the genetic improvement of such complex traits as disease resistance. This state-of-the-art methodology for modern selective breeding schemes in aquaculture has an advantage over conventional breeding, in that it can accelerate the breeding process, thus reducing costs and obtaining higher selection accuracy [44]. To enhance genomic selection, a reference population occurs in which the animals are genotyped and recorded for the trait of interest to train the genomic selection model and estimate the effect of each SNP allele with statistical models [44,45,46]. The SNP effects are then used to estimate the genetic value for the trait of the fish, without the need for phenotype recording. The results from an artificial challenge experiment can be extended to a much wider population.

For aquaculture species, the benefit of genomic selection is that it exploits family genetic variation for traits that cannot be measured directly on selection candidates [47,48]. Different applications of genomic selection have been described in salmonids. Vallejo et al. [44], for example, found genomic selection to be an efficient strategy for improving genetic resistance against BCWD in rainbow trout aquaculture. Genomic selection was also used in Atlantic salmon to predict breeding values for resistance to sea lice, a parasite that causes considerable economic loss [48,49].

Genomic selection has been used as a good alternative to increase the resistance of striped catfish to bacillary necrosis of Pangasius, a severe disease caused by *Edwardsiella ictalurid* [50]. Zhang et al. [51] reported the application of genomic selection to improve the prediction veracity of columnaris disease in catfish.

Finally, Lillehamer et al. [52] reported on genomic selection as a strategy for controlling WSSV and *Vibrio parahaemolyticus* outbreaks in shellfish (*Litopenaeus vannamei*). However, genotyping costs are a major limitation to implementing genomic selection.

### 3.4. Genome Editing

Genome editing is a novel breeding technology. Genome editing with CRISPR/Cas9 or TALENs technologies can accelerate genetic gain in the breeding of most aquaculture species by the rapid introduction of favorable changes in candidate genes into the genomes. Three main approaches to improving disease resistance through genome-editing are distinguished: (1) removing or fixing functional alleles segregation within broodstock populations in a selective breeding program; (2) introgression of a favorable variant from different populations; (3) creation of favorable alleles de novo [1]. CRISPR/Cas9 was recently applied to the aquaculture production of several salmonid species, Pacific oysters, Nile tilapia, and gilthead sea bream [53,54,55,56]. Debate surrounds the definition of genetic modification and whether genome editing should be considered as part of it, and therefore regulated with stringent rules or not. Uncertainty and differences exist between countries about genome editing regulations. Providing the general public and stakeholders with reliable information about the risks and benefits of genome editing holds a key role for speeding up its acceptance [1,57].

### 3.5. Novel Approaches

Machine learning applied to genetics involves data-driven systems that recognize certain data input patterns and improve genomic prediction accuracies for complex traits, especially for disease resistance in aquaculture species [49]. Abdelrahaman et al. [58] reported the use of machine learning to classify transcriptome and proteome data by pattern recognition in an analytical bioinformatic approach in which the modelling of expression patterns of genes can single out those responsible for a certain trait or response.

Different approaches to improve the accuracy of genomic selection include genotype imputation, genomic prediction, and increased automation. In Atlantic salmon, for example, genomic selection achieves high accuracy and performance but not when the relationship between training and validation animals becomes more distant, so a major challenge is to improve prediction accuracy in distant relatives by identifying the functional variants that impact on desired traits. Improving prediction accuracy across generations could also be a valid approach in rainbow trout, in conjunction with improved knowledge of the functional variants that impact on traits of commercial interest. New genomic selection strategies in catfish could exploit the genotyping array of a sufficient number of individuals without increasing population inbreeding. In crustaceans, the economic benefits of genomic selection versus the extra costs of genotyping need to be assessed, taking into account that a new genotyping technique, such as genotype imputation, might be more cost-effective [59].

Omics approaches, such as transcriptomics (gene expression mRNA level), proteomics (protein expression), and metabolomics (metabolite concentration), have become a powerful multidisciplinary tool for research because of their potential to unravel novel mechanisms for system biology.

Table 1 presents an overview of current genetic approaches.

## 4. Pathogen Characterization

Disease results from interactions between the host, the pathogen, and the environment. The host and the pathogen evolve mutually, but the underlying genetic mechanisms are still poorly understood [15]. Pathogen evolution plays a central role in the emergence and spread of disease in aquaculture. Mutual adaptation and counter-adaptation co-occur in the interaction between host and pathogen, to reach a compromise between pathogen dissemination and damage to the host. Changes in pathogens over time lead to a loss of efficacy of treatment and of vaccines, as the pathogen acquires greater resistance and virulence.

The use of antibiotics in aquaculture induces antimicrobial resistance through the horizontal gene transfer of antibiotic-resistant genes and of mobile elements in the pathogens and the surrounding bacteria in the water, sediment, and fish commensal strains. Environmental pressure, e.g., climate change, has a role in antibiotic resistance: rising temperatures affect bacterial cell physiology in the same way as antibiotics [60].

Studies on the evolution of vaccine resistance have focused on the spread of escape mutants that display epitopes different from those in the vaccine and thus escape immune recognition and increase pathogen virulence [61]. Conventional disease control methods, including breeding for resistance, have been identified as potential drivers of virulence in aquaculture [8]. Selective breeding strategies in the host need to be purposely informed by pathogen characterization over time.

A host’s ability to contain and resist pathogens over time depends on its genetic resistance and pathogen virulence. When a single gene is responsible for host resistance, selection is quite simple, but defense may be effective only in the short term. To illustrate: the host and the pathogen run a race where the host struggles to resist the pathogen and the pathogen overcomes the host’s resistance. This single gene race is quickly won by the pathogen because it has a much shorter generation time and a much faster evolutionary speed. In contrast, overcoming multigenic resistance is more difficult for the pathogen as it needs to overcome multiple barriers. Accordingly, strategies such as gene pyramiding are based on the introgression of multiple resistance genes into a pathogen by backcross or genetic engineering [62].

Genetics and genomics in the evaluation of pathogen evolution by molecular characterization, virulence, and antibiotic-resistant gene characterization are useful tools for the application of mitigation strategies.

### 4.1. Conventional Methods

Pathogen identification and diagnosis were largely based on conventional culture and biochemical analysis. Despite their numerous advantages, these methods are very time consuming and laborious. Furthermore, some microorganisms cannot be cultivated in the laboratory or are difficult to grow.

At the same time, virulence factors have traditionally been studied in vivo. While in vivo assays remain in many cases, irreplaceable, research continues to seek alternatives in line with the 3Rs principle of replacement, reduction, and refinement in experimental procedures.

The Kirby–Bauer disk diffusion susceptibility test and minimum inhibitory concentration (MIC) detection are standardized techniques for *testing* the antimicrobial resistance of rapidly growing pathogens.

### 4.2. Molecular Methods

Advances in molecular methods have provided a novel set of tools that characterize pathogens in ever greater detail. The introduction of PCR revolutionized diagnostic methods for the characterization of infectious disease agents, virulence, and antibiotic-resistant genes. Real-time PCR has improved the speed, sensitivity, and specificity of testing, in addition to providing quantitative results [63].

Molecular methods for the characterization of microorganisms and of relationships between isolates may be classified into three main groups: PCR-based, restriction enzymes-based, and sequencing-based. PCR-based methods (AFLP, RAPD, VNTR) [64,65,66] (Box 1) are commonly used for pathogen typing and as genetic markers for mapping analysis in aquaculture species. The development of PCR-based amplified fragment length polymorphism (AFLP) analysis is a key advancement in simple typing techniques. In this automated method two restriction endonucleases are used in combination with one set of PCR primers. Another powerful method for typing bacterial species is random amplified polymorphic DNA (RAPD), a widely used PCR technique for subtyping bacterial pathogens, where amplified DNA segments are chosen randomly.

Repetitive-sequence-based PCR typing is another subtyping technique that utilizes PCR: repetitive elements are randomly distributed throughout the bacterial genome; the primers complementary to these repeats are used to amplify the differently sized DNA fragments lying between these repeats [67].

Multiple locus variable number of tandem repeats analysis (MLVA) involves the rapid determination of the number and length of a variable number of tandem repeats (VNTRs) to generate a DNA fingerprint or a bacterial isolate.

Pulsed-field gel electrophoresis (PFGE) and restriction fragment length polymorphism (RFLP) analysis are considered gold standard genotyping methods based on the use of restriction enzymes. For example, the genome is cut with a rare-cutter enzyme in PFGE, and a gel band pattern is visualized using a hexagonal electrode (CHEF) system to resolve large size bands. The technique provides for unique fingerprinting profiles. It is one of the most widely used molecular typing strategies for the molecular characterization of numerous pathogenic microorganisms and a powerful method for epidemiological studies [68]. RFLP exploits differences in nucleotides recognized by frequent-cutter restriction enzymes to compare various DNA molecules [69].

The rapid accumulation of genetic sequence data in databanks led to the development of multilocus sequence typing (MLST), a new technique based on the sequencing of several housekeeping genes dispersed throughout the genome and characterized by SNPs. The results are coded into a numbering system for easy portability and comparison. This technique is an essential tool for population studies of pathogenic microorganisms [70]. Its main advantages are high discriminatory power and standardization as achieved with PFGE, but in less time.

Table 2 presents typing techniques in relation to accuracy, time, and costs.

### 4.3. Whole Genome Sequencing (WGS)

Whole genome sequencing (WGS) of bacterial and viral pathogens is the most recent development in characterization techniques. It identifies sequence fragments specific to the taxonomic level. With access to the full genetic content of microorganisms, rational selection of DNA fragments has enabled the creation of a wide array of detection and typing methods, as well as specialized tools that can identify and characterize genetic markers, such as the genes associated with antibiotic resistance or virulence factors. The study of resistant and susceptible strains and their assimilation has shed light on the resistome, which is the set of genetic markers associated with antibiotic resistance [71].

The development of next generation sequencing (NGS) platforms, together with decreasing costs for sequencers and reagents and increasing speed and discriminatory power, has enabled applications to generate data on genes within a bacterial genome from large population samples and to identify virulence and antimicrobial-resistance mechanisms.

## 5. Conclusions

Breeding programs based on genetic resistance traits have great potential to support the development of an efficient and sustainable aquaculture. Advances in technologies, such as sequencing, genomics and bioinformatics, have aided in optimizing selection and pathogen characterization and pathogen control and mitigation strategies. The increases in aquaculture efficiency, profitability, and sustainability have helped to reduce its environmental footprint. Aquaculture may be set to meet the global protein demand in human nutrition. Although half of total fish production is derived from aquaculture, the market is becoming a sector of small-scale producers no longer dominated by large companies: about 90% of output is from low- and middle-income countries [14].

Moreover, despite the rapid growth in aquaculture production, only 10% of stock is genetically enhanced [12] and progress differs noticeably for different aquaculture species. Breeding in salmonid species has progressed faster than in other species [71] and can be used as a model to obtain genetic advances in diverse aquaculture productions. The reasons for this low percentage of improved stock are manifold: size and stage of development of industries; inefficient control of reproduction; number of species farmed; difficulty in retaining pedigree throughout the production process; inability to obtain large phenotypic data sets; and lack of information about genetic parameters for traits [12,72].

MAS was developed to aid fish selection and remains a useful tool for traits related to QTL of large effect, while there are limited applications for complex traits related to genes of smaller effect. In such cases, genomic selection approaches need to be developed.

The genetic response of breeding programs can be refined by genomic selection. Higher accuracy of selection and subsequent higher rates of genetic gain (up to 10% by body weight) can be achieved in comparison to conventional selection [45,73,74]. The reduction of generation intervals by selecting candidates early in life based on their genomic breeding value can be exploited to improve genetic gain [74,75]. Furthermore, genomic selection can reduce inbreeding rates by up to 81% in comparison to conventional selection [76]. Rapid progression in selection by reducing the generation interval could yield additional gain to counteract pathogen evolution.

Priority-setting strategies, together with cost–benefit analysis, can guide informed decisions about the best breeding strategy to apply.

Public/private partnerships in innovation should be implemented to develop technologies and translate them into novel, low-cost commercial applications, affordable for all, and especially for small-scale farmers. The use of low-cost genome sequencing enables large scale, cost-effective genotyping, which is particularly important for species without a reference genome or access to commercial genotyping arrays. This would enable the wider application of genetics and genomics approaches to aquaculture.

## Figures and Tables

**Table 1:** Genetic selection in aquaculture species.

Genetic Approach	Species	Pathogen/Disease	Reference
Marker-assisted Selection	Rainbow trout	*Rhabdovirus*	[24]
	Rainbow trout	*Aeromonas* *salmonicida*	[25]
	Rainbow trout	*Vibrio* *anguillarum*	[26]
	Rainbow trout	*Flavobacterium psychrophilum*	[27]
	Rainbow trout	Viral hemorrhagic septicemia virus	[27]
	Rainbow trout	*Flavobacterium columnare*	[28]
	Rainbow trout	*Flavobacterium psychrophilum*	[29]
	Atlantic salmon	Infectious Pancreatic Necrosis Virus	[22,23]
	Atlantic salmon	*Caligus rogercresseyi*	[31]
	Atlantic salmon	*Neoparamoeba perurans*	[30]
	Atlantic salmon	*Lepeophtheirus salmonis*	[32]
	Shrimp	White spot syndrome virus	[33]
Gene-assisted Selection	Atlantic salmon	Infectious pancreatic necrosis virus	[34]
	Atlantic salmon	Infectious pancreatic necrosis virus	[35]
	Rainbow trout	*Lactococcus garvieae*	[36]
	Rainbow trout	*Flavobacterium psychrophilum*	[37]
	Atlantic salmon	Infectious salmon anaemia	[38]
	Atlantic salmon	*Aeromonas salmonicida*	[39]
	Oyster	*Perkinsus marinus*	[41]
Genomic Selection	Rainbow trout	*Flavobacterium psychrophilum*	[44]
	Shrimp–Oyster	*-*	[46]
	Sea bass–Sea bream	*-*	[47]
	Atlantic salmon	*Caligus rogercresseyi*	[49]
	Atlantic salmon	*-*	[48]
	Atlantic salmon	*Lepeophtheirus salmonis*	[49]
	Atlantic salmon	*-*	[52]
	Atlantic salmon	*Neoparamoeba perurans*	[30]
	Catfish	*Edwardsiella ictaluri*	[50]
	Catfish	*Flavobacterium columnare*	[51]
	Shrimp	White spot syndrome virus	[52]
Genome Editing	Sea bream	*-*	[54]
	Catfish	*-*	[55]

**Table 2:** Comparison of common typing techniques in relation to accuracy, time, and costs.

Typing Technique	Repeatability	Reproducibility	Time (Days)	Cost
MLST	high	high	3+	medium–high
PFGE	medium–high	medium–high	3	high
RFLP	medium–high	medium	1–3	medium
AFLP	high	medium–high	2	low–medium

## Data Availability

Not applicable.

## References

[1] Gratacap R., Wargelius A., Edvardsen R.B., Houston R.D. (2019). Potential of Genome Editing to Improve Aquaculture Breeding and Producton. Trends Genet..

[2] API (2022). Associazione Piscicoltori Italiani—Acquacoltura.Org—Elaborazione Dati. https://www.acquacoltura.org.

[3] Moreira M., Schrama D., Farinha A.P., Cerqueira M., de Magalhaes C.R., Carrilho R., Rodrigues P. (2021). Fish Pathology Research and Diagnosis in Aquaculture of Farmed Fish: A Proteomic Perspective. Animals.

[4] Altizer S., Dobson A., Hosseini P., Hudson P., Pascual M., Rohani P. (2006). Seasonality and the dynamics of infectious diseases. Ecol. Lett..

[5] Cascarano M.C., Stavrakidis-Zachou O., Mladineo I., Thompson K.D., Papandroulakis N., Katharios P. (2021). Mediterranean Aquaculture in a Changing Climate: Temperature Effects on Pathogens and Diseases of Three Farmed Fish Species. Pathogens.

[6] Sommerset I., Krossoy B., Biering E., Frost P. (2005). Vaccines for fish in aquaculture. Expert Rev. Vaccines.

[7] Adams A. (2019). Progress, challenges and opportunities in fish vaccine development. Fish Shellfish Immunol..

[8] Kennedy D.A., Kurath G., Brito I.L., Purcell M.K., Read A.F., Winton J.R., Wargo A.R. (2015). Potential drivers of virulence evolution in aquaculture. Evol. Appl..

[9] Novais C., Campos J., Freitas A.R., Barros M., Silveira E., Coque T.M., Antines P., Peixe L. (2018). Water supply and feed as sources of antimicrobial-resistant *Enterococcus* spp. in aquacultures of rainbow trout (*Oncorhyncus mykiss*), Portugal. Sci. Total Environ..

[10] Heuer O.E., Kruse H., Grave K., Collignon P., Karunasagar I., Angulo F.J. (2009). Human Health Consequences of Use of Antimicrobial Agents in Aquaculture. Clin. Infect. Dis..

[11] Gjedrem T., Baranski M. (2009). Selective Breeding in Aquaculture: An Introduction.

[12] Gjedrem T., Robinson N., Rye M. (2012). The importance of selective breeding in aquaculture to meet future demands for animal protein: A review. Aquaculture.

[13] Gjedrem T., Rye M. (2018). Selection response in fish and shellfish: A review. Rev. Aquac..

[14] Houston R.D., Bean T.P., Macqueen D.J., Gundappa M.K., Jin Y.H., Jenkins T.L., Selly S.L.C., Martin S.A.M., Stevens J.R., Santos E.M. (2020). Harnessing genomics to fast-track genetic improvement in aquaculture. Genetics.

[15] Masri L., Branca A., Sheppard A.E., Papkou A., Laehnemann D., Guenther P.S., Prahl S., Saebelfeld M., Hollensteiner J., Liesegang H. (2015). Host-Pathogen Coevolution: The selective advantage of Bacillus thuringiensis virulence and its cry toxin genes. PLoS Biol..

[16] Gjedrem T. (2010). The first family-based breeding program in aquaculture. Rev. Aquac..

[17] Odegard J., Baranski M., Gjerde B., Gjedrem T. (2011). Methodology for genetic evaluation of disease resistance in aquaculture species: Challenges and future prospects. Aquac. Res..

[18] Guy D.R. (2011). Genetic Resistance to Infectious Pancreatic Necrosis Virus in Pedigreed Atlantic Salmon (Salmo salar).

[19] Storset A., Strand C., Wetten M., Kjoglum S., Ramstad A. (2007). Response to selection for resistance against infectious pancreatic necrosis in Atlantic salmon (*Salmo salar* L.). Aquaculture.

[20] Yanez J.M., Houston R.D., Newman S. (2014). Genetics and genomics of disease resistance in salmonid species. Front. Genet..

[21] Houston R.D. (2017). Future directions in breeding for disease resistance in aquaculture species. Braz. J. Anim. Sci..

[22] Houston R.D., Gheyas A., Hamilton A., Guy D.R., Tinch A.E., Taggart J.B., McAndrew B.J., Haley C.S., Bishop S.C. (2008). Detection and confirmation of a major QTL affecting resistance to infectious pancreatic necrosis (IPN) in Atlantic salmon (*Salmo salar*). Dev. Biol..

[23] Moen T., Baranski M., Sonesson A.K., Kjoglum S. (2009). Confirmation and fine-mapping of a major QTL for resistance to infectious pancreatic necrosis in Atlantic salmon (*Salmo salar*): Population-level associations between markers and trait. BMC Genom..

[24] Verrier E.R., Dorson M., Mauger S., Torhy C., Ciobotaru C., Hervet C., Dechamp N., Genet C., Boudinot P., Quillet E. (2013). Resistance to a rhabdovirus (VHSV) in rainbow trout: Identification of a major QTL related to innate mechanisms. PLoS ONE.

[25] Marana M.H., Karami A.M., Odegard J., Zuo S., Jaafar R.M., Mathiessen H., Jorgensen L.V.G., Kania P.W., Dalsgoard I., Nielsen T. (2021). Whole-genome association study searching for QTL for *Aeromonas salmonicida* resistance in rainbow trout. Sci. Rep..

[26] Karami A.M., Odegard J., Marana M.H., Zuo S., Jaafar R., Mathiessen H., Jorgensen L., Von G., Kania P.W., Dalsgaard I. (2020). A major QTL for resistance to *Vibrio anguillarum* in Rainbow Trout. Front. Genet..

[27] Vallejo R.L., Palti Y., Lui S., Evenhuis J.P., Gao G., Rexroad C.E., Wiens G.D. (2014). Detection of QTL in Rainbow Trout affecting survival when challenged with *Flavobacterium psychrophilum*. Mar. Biotechnol..

[28] Evenhius J.P., Leeds T.D., Marancik D.P., Lapatra S.E., Wiens G.D. (2015). Rainbow trout (*Oncorhynchus mykiss*) resistance to columnaris disease is heritable and favorably correlated with bacterial cold water disease resistance. J. Anim. Sci..

[29] Silva R.M.O., Evenhuis J.P., Vallejo R.Ç., Tsuruta S., Wiens G.D., Martin K.E., Parsons J.E., Palti Y., Lourenco D.A.L., Leeds T.D. (2019). Variance and covariance estimates for resistance to bacterial cold water disease and columnaris disease in two rainbow trout breeding populations. J. Anim. Sci..

[30] Robledo D., Matika O., Hamilton A., Houston R.D. (2018). Genome-wide association and genomic selection for resistance to Amoebic Gill Disease in Atlantic salmon. G3 Genes Genomes Genet..

[31] Robledo D., Gutierrez A.P., Barria A., Lhorente J.P., Houston R.D., Yanez J.M. (2019). Discovery and functional annotation of Quantitative Trait Loci affecting resistance to Sea Lice in Atlantic Salmon. Front. Genet..

[32] Jones S. (2013). Mechanisms of resistance among salmon to the parasitic copepod *Lepeophtheirus salmonis*. J. Aquac. Res. Dev..

[33] Robinson N.A., Gopikrishna G., Baranski M., Katheni V.K., Shekhar M.S., Shanmugakarthik J., Jothivel S., Gopal C., Ravichandran P., Gitterle T. (2014). QTL for white spot syndrome virus resistance and the sex-determining locus in the Indian black tiger shrimp (Penaeus Monodon). BMC Genom..

[34] Moen T., Torgersen J., Santi N., Davidson W.S., Baranski M., Odegard J., Kjoglum S., Velle B., Kent M., Lubieniecki K.P. (2015). Epithelial cadherin determines resistance to infectious pancreatic necrosis virus in Atlantic Salmon. Genetics.

[35] Pavelin J., Jin Y.H., Gratacap R., Taggart J.B., Hamilton A., Verner-Jeffreys D.W., Paley R.K., Rubin C.J., Bishop S.C., Bron D.E. (2021). The need-8 activating enzyme gene underlies genetic resistance to infectious pancreatic necrosis virus in Atlantic salmon. Genomics.

[36] Colussi S., Prearo M., Bertuzzi A., Scanzio T., Peletto S., Favaro L., Modesto P., Maniaci M.G., Ru G., Desiato R. (2015). Association of a specific major histocompatibility complex class IIb single nucleotide polymorphism with resistance to lactococcus in rainbow trout, *Oncorhyncus mykiss* (Walbaum). J. Fish Dis..

[37] Johnson N.A., Vallejo R.L., Silverstein J.T., Welch T.J., Wiens G.D., Hallerman E.M., Palti Y. (2008). Suggestive association of major histocompatibility IB genetic markers with resistance to bacterial cold-water disease in rainbow trout (*Oncorhynchus mykiss*). Mar. Biotechnol..

[38] Kjoglum S., Larsen S., Bakke H.G., Grimholt U. (2006). How specific MHC classI and class II combinations affect disease resistance against infectious salmon anaemia in Atlantic salmon (*Salmo salar*). Fish Shellfish Immunol..

[39] Kjoglum S., Larsen S., Bakke H.G., Grimholt U. (2008). The effect of specific MHC class I and class II combinations on resistance to furunculosis in Atlantic salmon (*Salmo salar*). Scand. J. Immunol..

[40] Miller H.C., Lambert D.M. (2004). Gene duplication and gene conversion in class II MHC genes of New Zealand robins (Petroicidae). Immunogenetics.

[41] He Y., Yu H., Bao Z., Zhong Q., Guo X. (2012). Mutation promoter region of a serine protease inhibitor confers *Perkinsus marinus* in the eastern oyster (*Crassostrea virginica*). Fish Shellfish Immunol..

[42] Goddard M., Hayes B.J., Meuwissen T.H.E. (2010). Genomic selection in livestock populations. Genet. Res..

[43] Tsairidou S., Hamilton A., Robledo D., Bron J.E., Houston R.D. (2020). Optimizing low-cost genotyping and imputation strategies for genomic selection in Atlantic salmon. G3 Genes Genomes Genet..

[44] Vallejo R.L., Silva R.M.O., Evenhuis J.P., Gao G., Lui S., Parson J.E., Martin K.E., Wiens G.D., Lourenco D.A.L., Leeds T.D. (2018). Accurate genomic predictions for BCWD resistance in rainbow trout are achieved using low-density SNP panels. Evidence that long-range LD is a major contributing factor. J. Anim. Breed. Genet..

[45] Wang T., Chen Y.P.P., MacLead I.M., Pryce J.E., Goddard M.E., Hayes B.J. (2017). Application of a Bayesan non-linear model hybrid scheme to sequence data for genomic prediction and QTL mapping. BMC Genom..

[46] Zenger K.R., Khatkar M.S., Jones D.B., Khalilisamani N., Jerry D.R., Raadsma H.W. (2019). Genomic selection in aquaculture: Application, limitations and opportunities with special reference to a marine shrimp and pearl oysters. Front. Genet..

[47] Griot R., Allal P., Brard-Fudulea S., Morvezen R., Haffray P., Francois Y., Morin T., Bestin A., Bruant J.S., Cariou S. (2021). Optimization of genomic selection to improve disease resistance in two marine fishes, the European sea bass (*Dicentrarchuslabrax*) and the gilthead sea bream (*Sparus aurata*). Front. Genet..

[48] Odegard J., Moen TSanti N., Koorsvoll S.A., Kjoglum S., Meuwissen T.H.E. (2014). Genomic prediction in an admixed population of Atlantic salmon (*Salmo salar*). Front. Genet..

[49] Tsai H.Y., Hamilton A., Tinch A.E., Guy D.R., Bron J.E., Taggart J.B., Gharbi K., Stear M., Matika O., Pong-Wong R. (2016). Genomic prediction of host resistance to sea lice in farmed Atlantic salmon populations. Genet. Sel. Evol..

[50] Vu N.T., Phuc T.H., Oah K.T.P., Sang N.V., TRang T.T., Nguyen N.H. (2022). Accuracies of genomic predictions for disease resistance of striped catfish to *Edwardsiellaictaluri* using artificial intelligence algorithms. G3 Genes Genomes Genet..

[51] Zhang Y., Lui Z., Li H. (2020). Genomic prediction of Columnaris disease resistance in catfish. Mar. Biotechnol..

[52] Lillehamer M., Bangera R., Salazar M., Vela S., Erazo E.C. (2020). Genomic selection for white spot syndrome virus resistance in whiteleg shrimp boosts survival under an experimental challenge test. Sci. Rep..

[53] Edvardsen R.B., Leininger S., Kleppe L., Skaftnesmo K.O., Wargelius A. (2014). Targeted mutagenesis in Atlantic Salmon (*Salmo salar* L.) using the CRISPR/Cas9 system induces complete knockout individuals in the F0 generation. PLoS ONE.

[54] Kishimoto K., Washio Y., Yoshiura Y., Tooda A., Ueno T., Fukuyama H., Kato K., Kinoshita M. (2018). Production of a breed of red sea bream *Pagrus major* with an increase of skeletal muscle mass and reduced body length by genome editing with CRISPR/Cas9. Aquaculture.

[55] Khalil K., Elayat M., Khalifa E., Daghash S., Elaswad A., Muller M., Abdelrahman H., Ye Z., Odin R., Drescher D. (2017). Generation of myostatin gene-edited channel catfish (*Ictalurus punctuatus*) via zygote injection of CRISPR/Cas9 system. Sci. Rep..

[56] Cleveland B.M., Yamaguchi G., Radler L.M., Shimizu M. (2018). Editing the duplicated insulin-like growth factor binding protein-2b gene in rainbow trout (*Oncorhynchus mykiss*). Sci. Rep..

[57] Mandrioli M. (2022). Genome editing among bioethics and regulatory practices. Biomolecules.

[58] Abdelrahman H., Elhady M., Alcivar-Warren A., Allen S., Al-Tobasei R., Bao L., Beck B., Blackbum H., Bosworth B., Buchana J. (2017). Aquaculture genomics, genetics and breeding in the United States: Current status, challenges, and priorities for future research. BMC Genom..

[59] Boudry P., Allol F., Aslam M.L., Bargelloni L., Bean T.P., Brard-Fudulea S., Brieuc M.S.O., Calboli F.C.F., Gilbey J., Haffray P. (2021). Current status and potential of genomic selection to improve selective breeding in the main aquaculture species of International Council for the Exploration. Aquac. Rep..

[60] Pepi M., Focardi S. (2021). Antibiotic-resistant bacteria in aquaculture and climate change: A challenge for health in the Mediterranean area. Int. J. Environ. Res. Public Health.

[61] Gandon S., Mackinnon M.J., Nee S., Read A.F. (2001). Imperfect vaccines and the evolution of pathogen virulence. Nature.

[62] Dormatey R., Sun C., Ali K., Coulter J.A., Bi Z., Bai J. (2020). Gene pyramiding for sustainable crop improvement against biotic and abiotic stresses. Agronomy.

[63] Kralik P., Ricchi M. (2017). A basic guide to Real Time PCR in microbial diagnostics: Definitions, parameters, and everything. Front. Microbiol..

[64] Sheeja T.E., Kumar I.P.V., Giridhari A., Minoo D., Rajesh M.K., Babu K.N. (2020). Amplified Fragment Length Polymorphism: Application and recent development. Mol. Plant Taxon..

[65] Babu K.N., Sheeja T.E., Minoo D., Rajesh M.K., Samsudeen K., Suraby E.J., Kumar I.P.V. (2021). Random Amplified Polyorphic DNA (RAPD) and derived techniques. Mol. Plant Taxon..

[66] Trees E.H., Lafon P., Vauterin P., Ribot E.M. (2010). Multilaboratory validation study of standardized multiple-locus variable-number tandem repeat analysis protocol for Shiga toxin-producing *Escherichia coli* O157: A novel approach to normalize fragment size data between capillary electrophoresis platforms. Foodborne Pathog. Dis..

[67] Healy M., Huong J., Bittner T., Lising M., Frye S., Raza S., Schrock R., Manry J., Renwick A., Nieto R. (2005). Microbial DNA typing by automated repetitive-sequence-based PCR. J. Clin. Microbiol..

[68] Neoh H.M., Tan X.E., Sapri H.F., Tan T.L. (2019). Pulsed-field gel electrophoresis (PFGE): A review of the “gold standard” for bacteria typing and current alternatives. Infect. Genet. Evol..

[69] Dai S., Long Y. (2015). Genotyping analysis using an RFLP assay. Plant Genotyping.

[70] Maiden M.C., Van Rensburg M.J.J., Bray J.E., Earle S.G., Ford S.A., Jolley K.A., McCarthy N.D. (2013). MLST revisited: The gene-by-gene approach to bacterial genomics. Nat. Rev. Microbiol..

[71] Fournier P.E., Dubourg G., Raoult D. (2014). Clinical detection and characterization of bacterial pathogens in the genomics era. Genome Med..

[72] You X., Shan X., Shi Q. (2020). Research advances in the genomics and applications for molecular breeding of aquaculture animals. Aquaculture.

[73] Jerry D., Purvis I., Piper L. (2001). Opportunities for genetic improvement in crustacean species. Proc. Assoc. Advmt. Anim. Breed. Genet..

[74] Campos-Montes G.R., Montaldo H.H., Martinez-Ortega A., Martinez Jimenez A., Castillo-Juarez H. (2013). Genetic parameters for growth and survival traits in Pacific white shrimp *Penaeus (Litopenaeus*) *vannamei* from a nucleus population undergoing a two-stage selection program. Aquac. Int..

[75] Castillo-Juarez H., Campos-Montes G.R., Caballero-Zamora A., Montaldo H.H. (2015). Genetic improvement of Pacific white shrimp [*Penaeus (Litopenaeus) vannamei*]: Perspectives for genomic selection. Front. Genet..

[76] Vandeputte M., Haffray P. (2014). Parentage assignment with genomic markers: A major advance for understanding and exploiting genetic variation of quantitative traits in farmed aquatic animals. Front. Genet..

